# Supplementary figures and images for: The importance of nonsense errors: Estimating the rates and implications of ribosome drop-off during protein synthesis
Source: PLoS Genet. 2026 Jun 9;22(6):e1012162. doi: 10.1371/journal.pgen.1012162 (PMC13271495; doi:10.1371/journal.pgen.1012162)

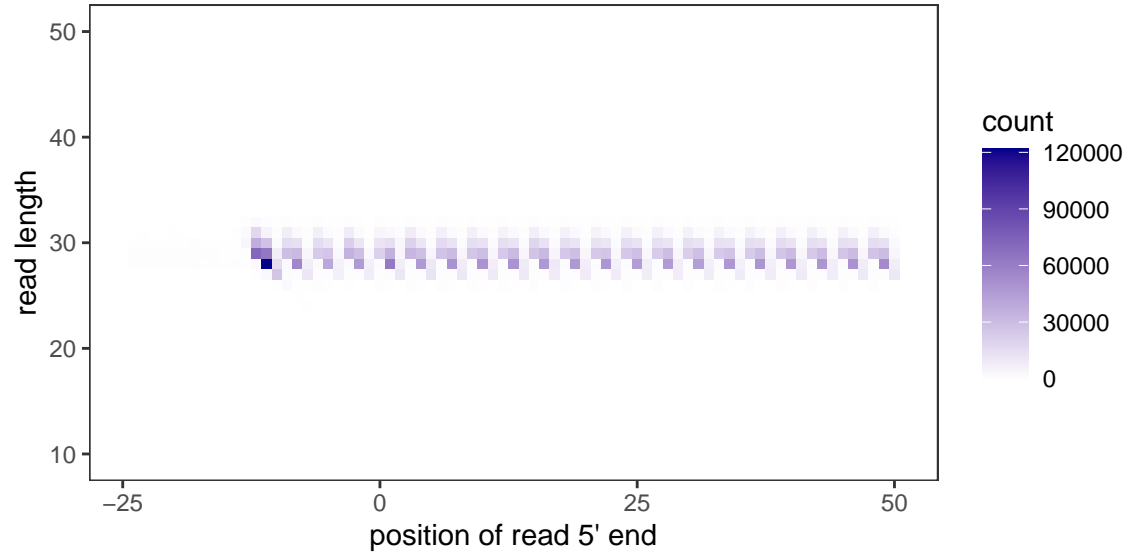

Supplement: S1 Fig — This illustrates the number of ribosome footprints assigned to nucleotide based on the 5’-end of the read. Darker colors indicate more ribosome footprints assigned to a nucleotide. The nucleotide at position 0 indicates the first nucleotide of the start codons. (PDF) [file pgen.1012162.s002.pdf]

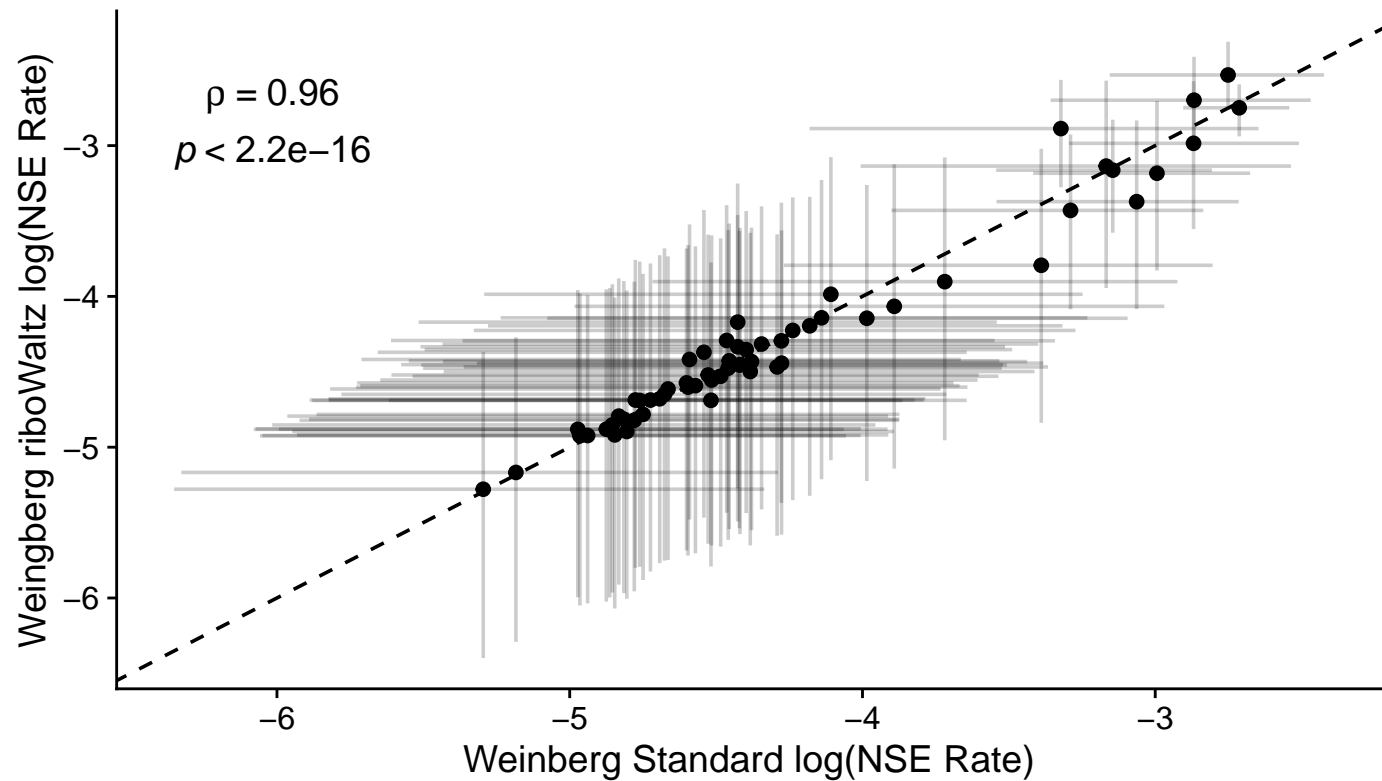

Supplement: S2 Fig — Comparison of NSE rate estimates from Weinberg et al. data using either the “standard” A-site 15 nt offsets vs. the offsets estimated by riboWaltz. Spearman rank correlation coefficient ρ is reported. (PDF) [file pgen.1012162.s003.pdf]

**A**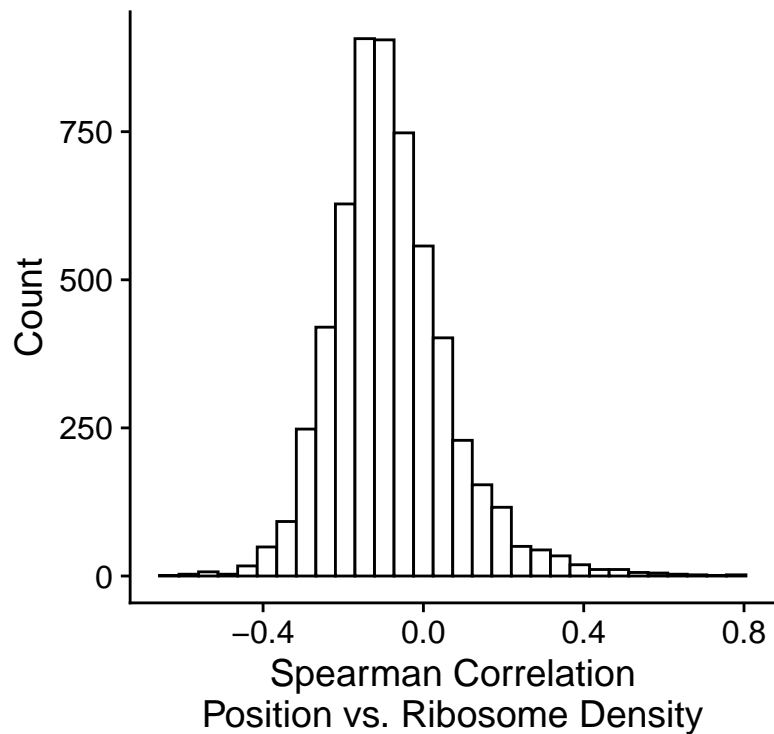**B****YDR512C**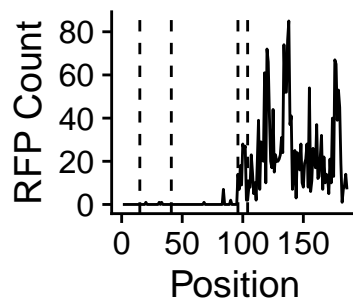**C****YGL220W**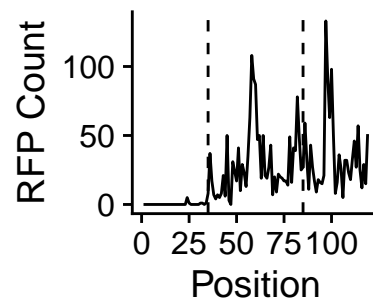**D****YER066W**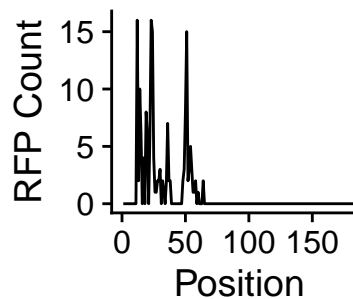**E****YIL009C-A**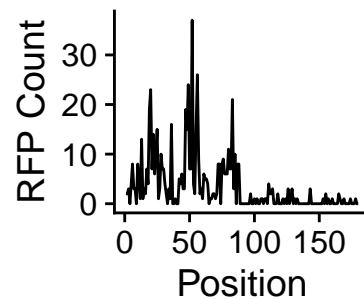

Supplement: S3 Fig — (A) Distribution of correlations between position within a gene and ribosome density. (B-C) Genes exhibiting sudden increase in ribosome densities. Dashed lines indicate ATG codons. (D-E) Genes exhibiting a sudden decrease in ribosome densities. (PDF) [file pgen.1012162.s004.pdf]

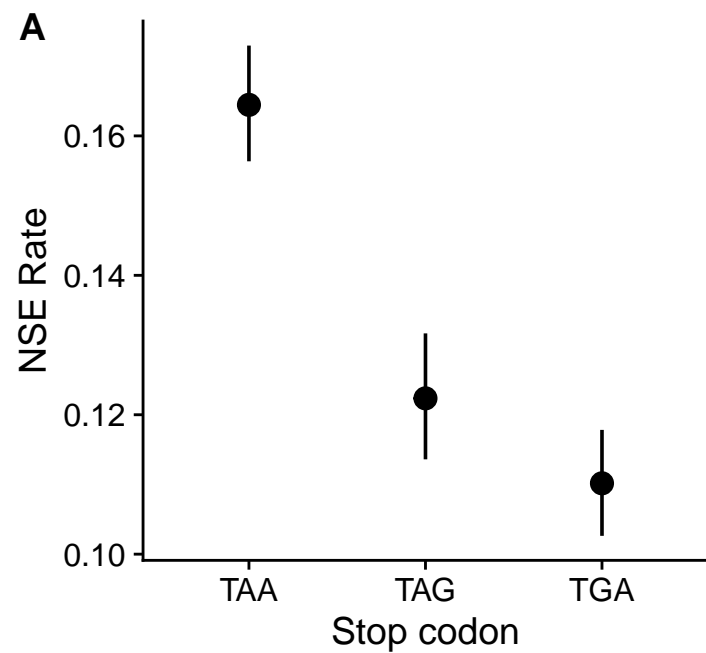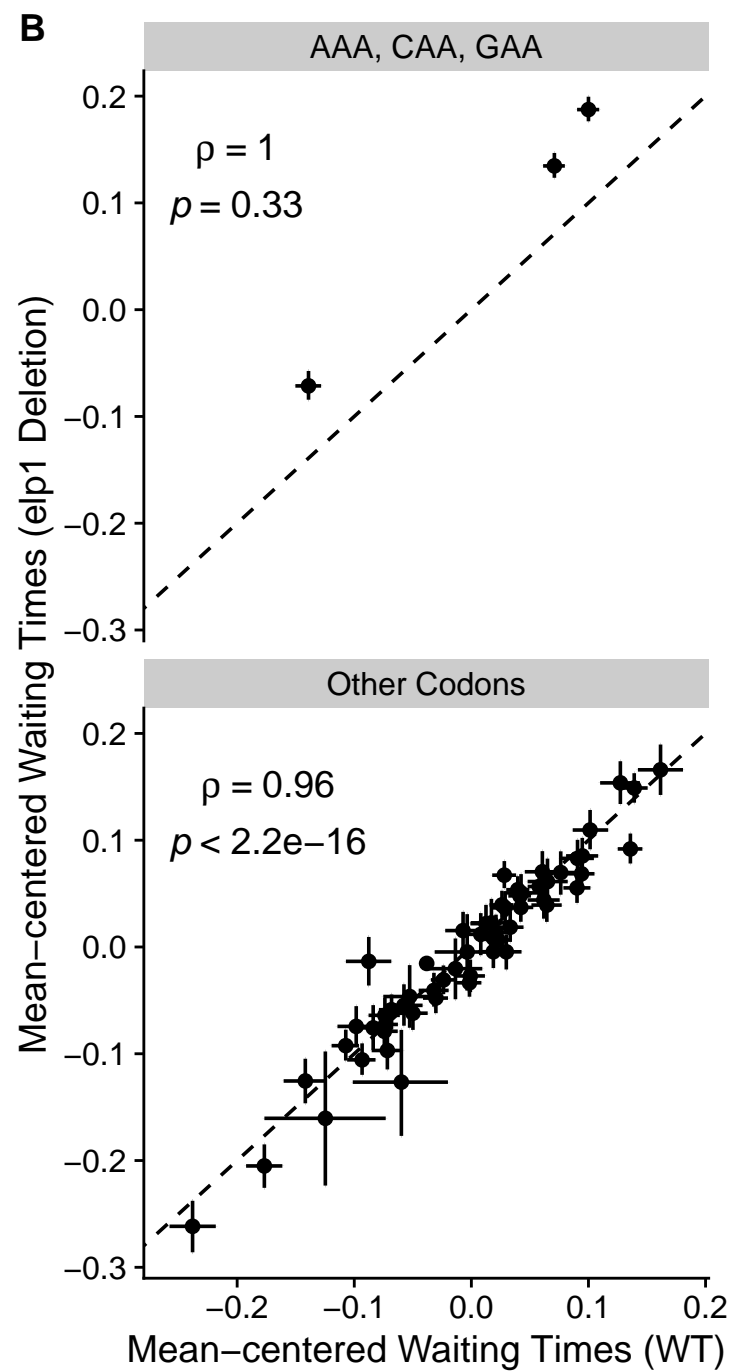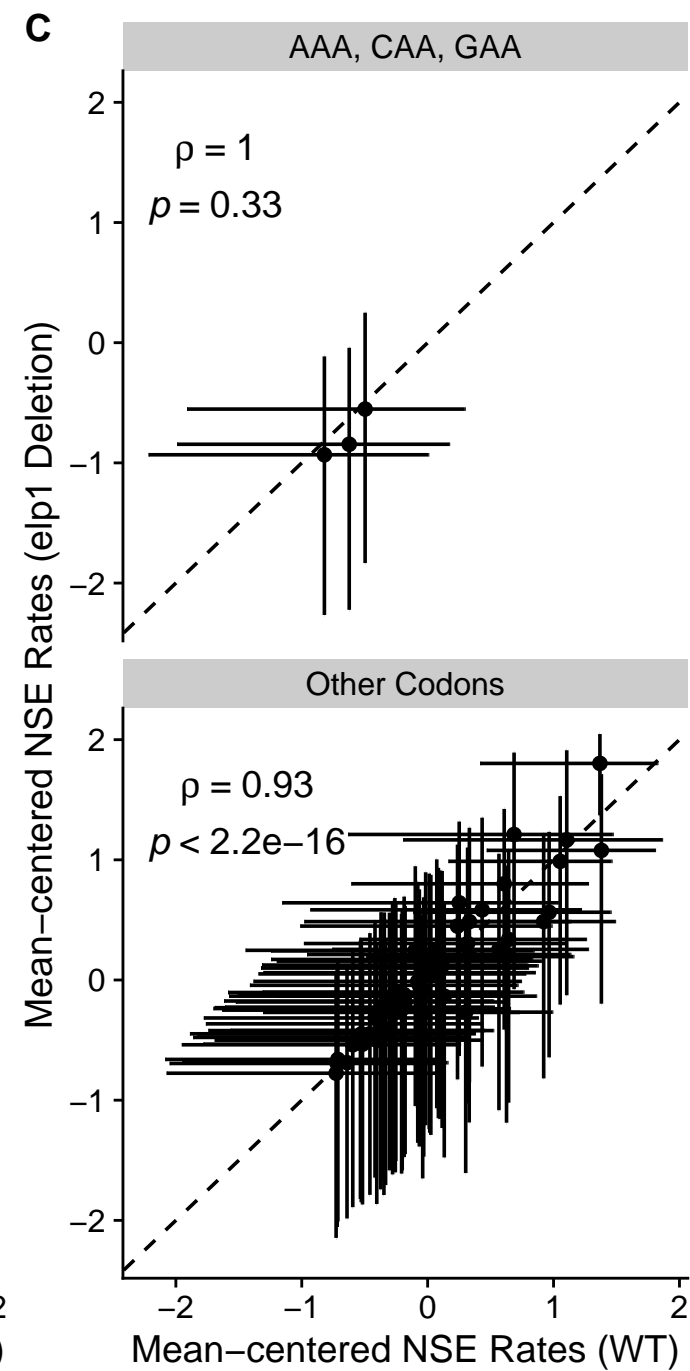

Supplement: S4 Fig — (A) Comparison of NSE rates b for stop codons. Data from Weinberg et al. was used, slightly modified to allow for up to 15 codons from empirically determined 3’-UTRs [54]. (B) Log fold-changes of mean-centered waiting times 1/c between the elp1 deletion strain and the reference wild-type strain obtained from [28]. Top panel indicates the codons known to be impacted by this tRNA modification enzyme deletion, while the bottom indicates the other 58 sense codons. (C) Same as in (B), but with the NSE rates b. (PDF) [file pgen.1012162.s005.pdf]

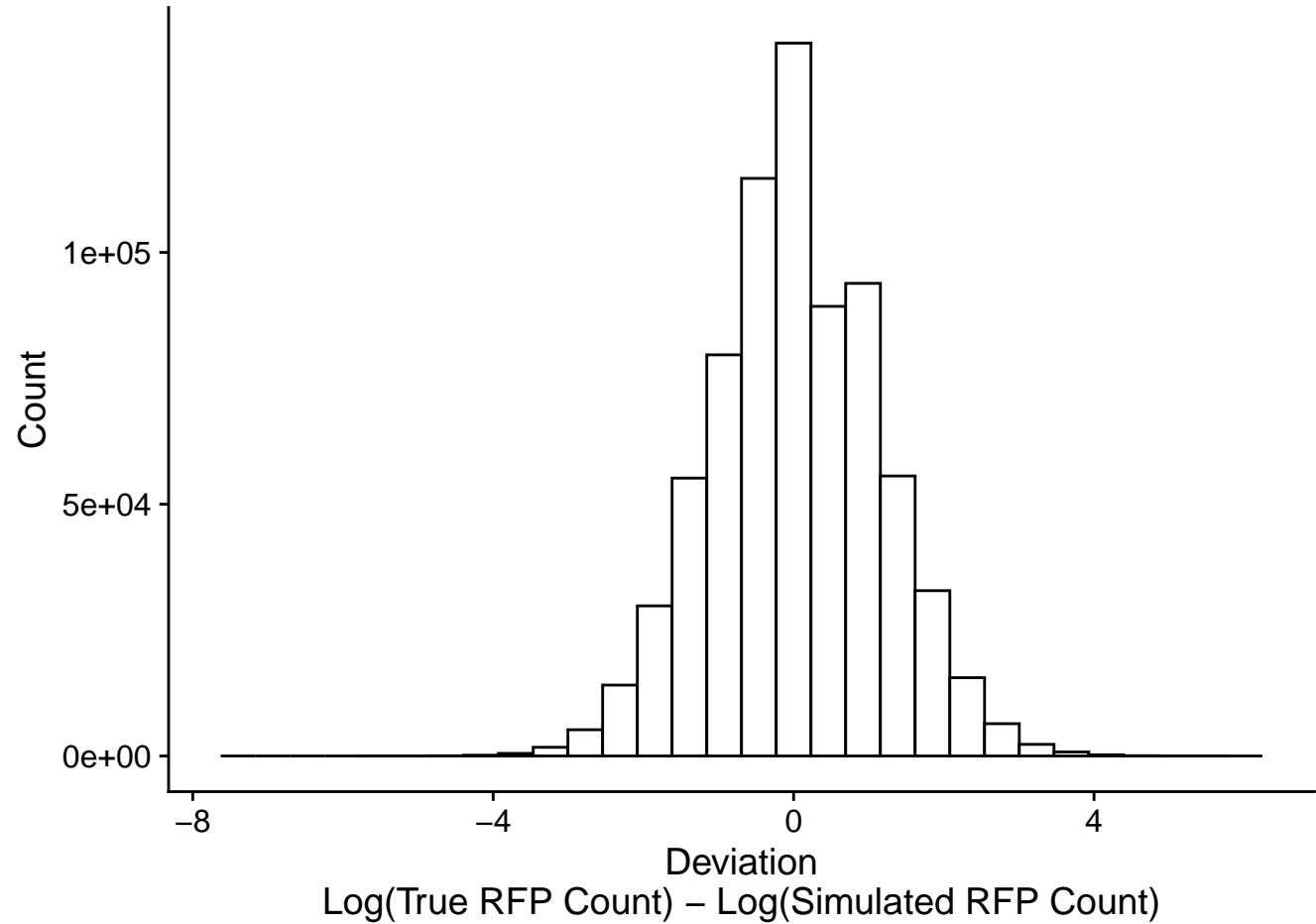

Supplement: S5 Fig — Deviations are calculated as the log fold-difference. (PDF) [file pgen.1012162.s006.pdf]

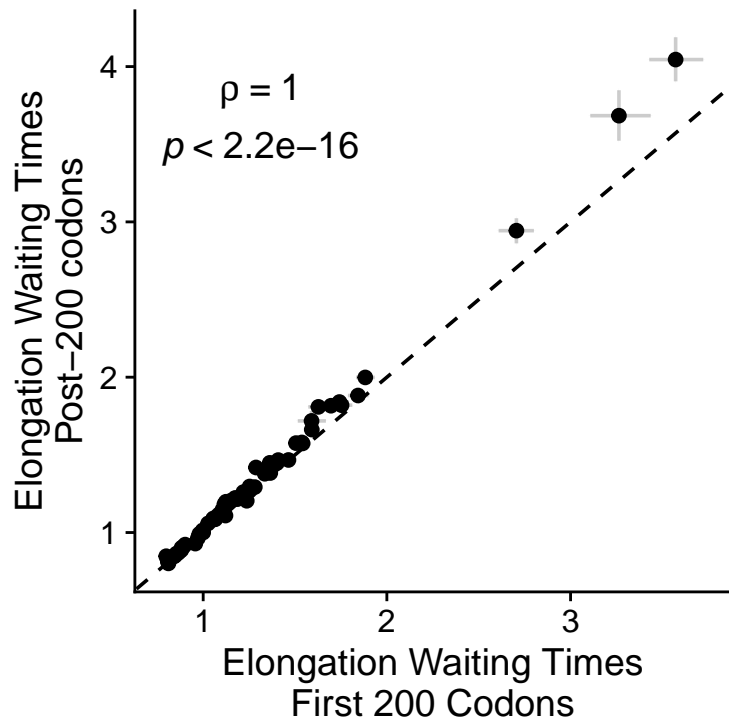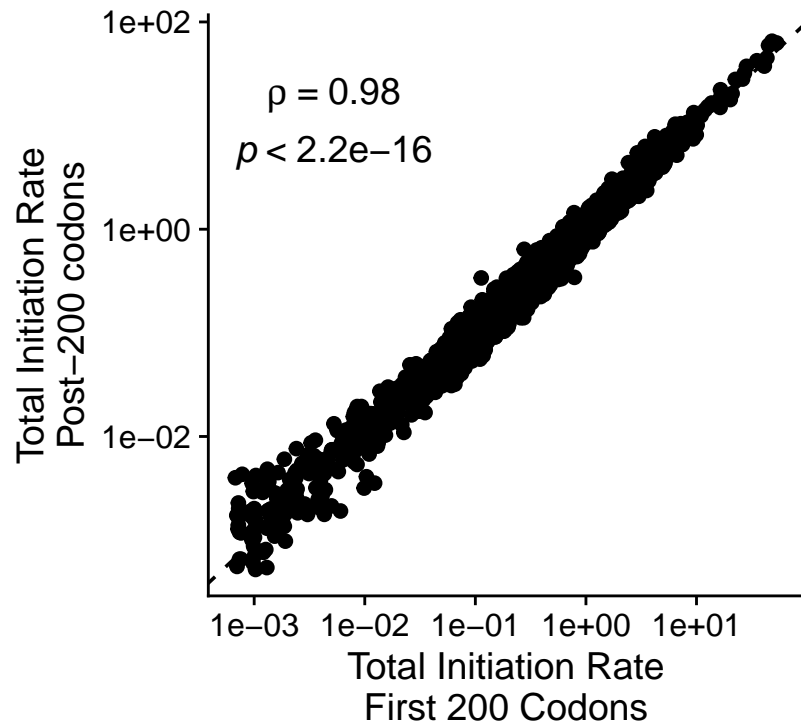

Supplement: S6 Fig — Comparison of (A) elongation waiting times and (B) total initiation rates when considering only the first 200 codons (i.e., the 5’-ramp region) vs. the remainder of the genes. Spearman rank correlations ρ are reported. Error bars represent 95% posterior probability intervals. (PDF) [file pgen.1012162.s007.pdf]

**A**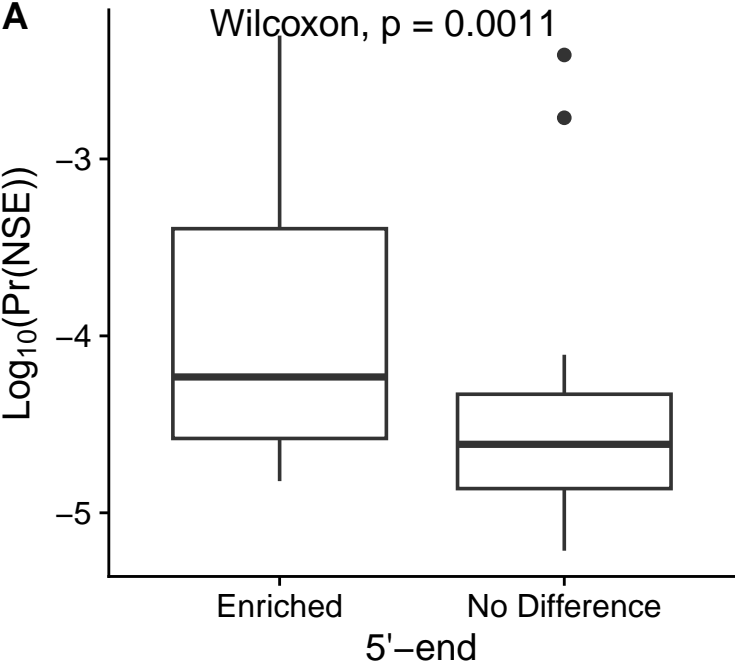**B**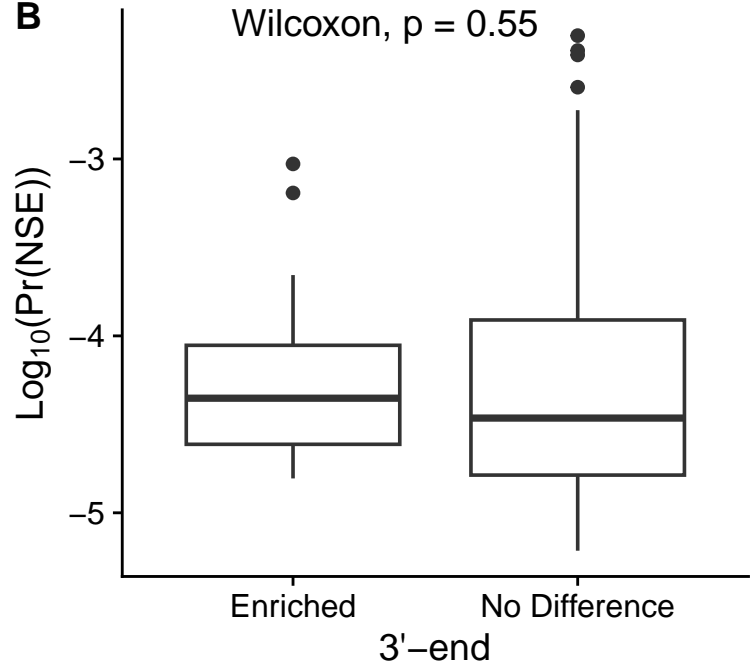

Supplement: S7 Fig — Difference in NSE probabilities between codons enriched in the (A) 5’-end and (B) 3’-ends of coding sequences (first and last 100 termini). Wilcoxon rank sum test p-values are reported. (PDF) [file pgen.1012162.s008.pdf]

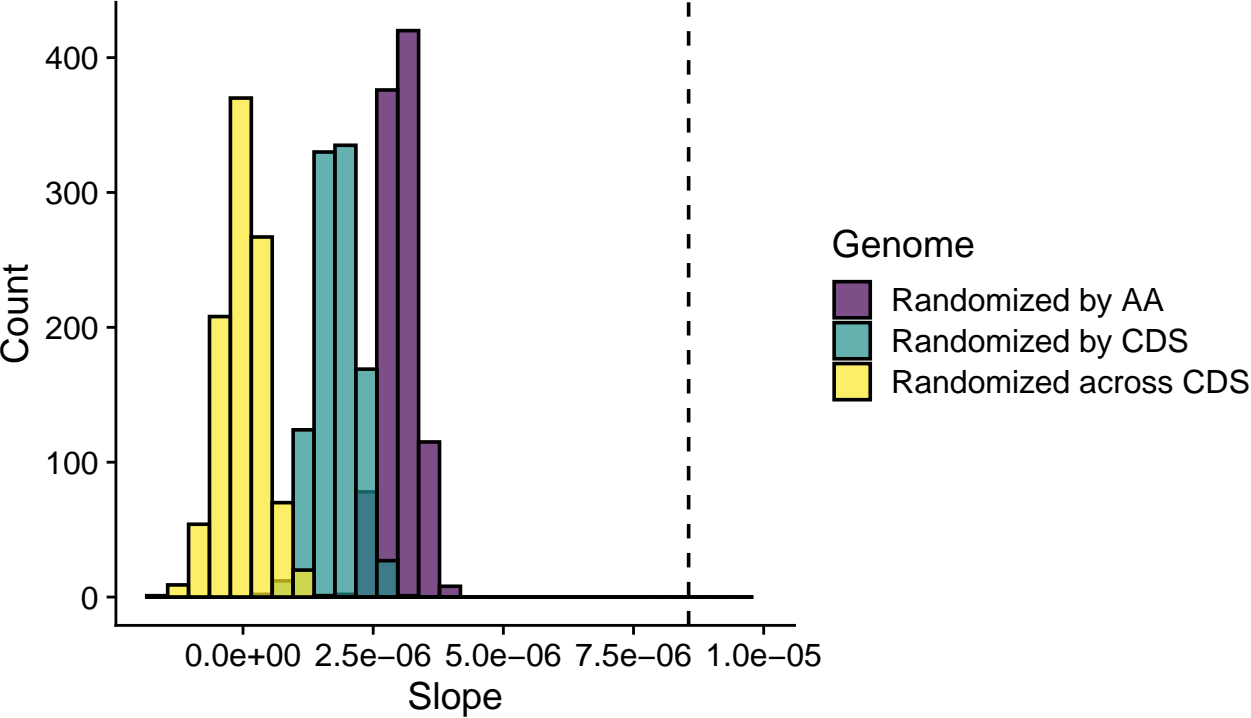

Supplement: S8 Fig — The slope for the real sequences is represented by the dashed line. (PDF) [file pgen.1012162.s009.pdf]

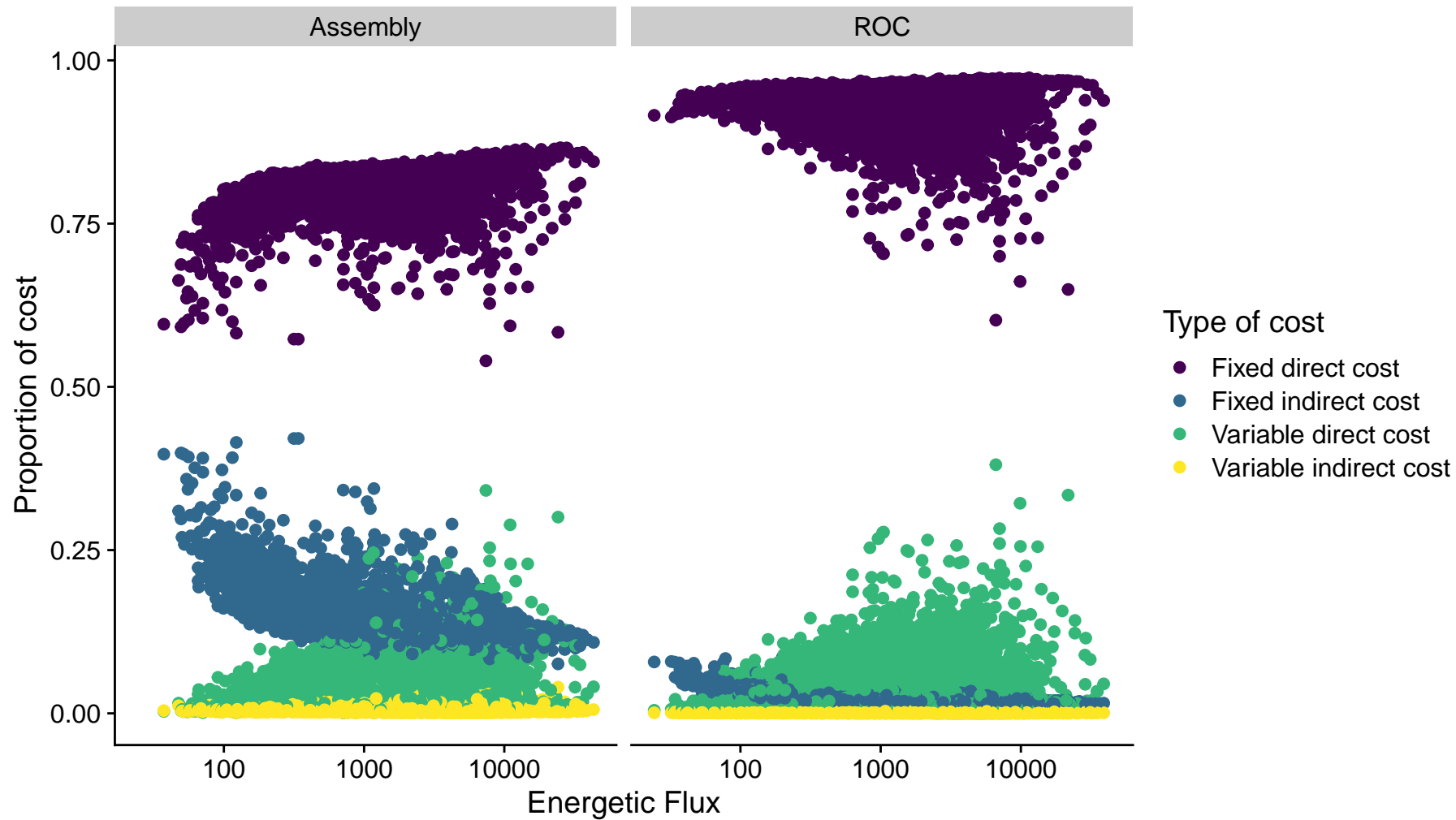

Supplement: S9 Fig — Comparison of proportion of total cost per gene as a function of total energetic flux (cost times the protein production rate) based on Ct1/2 (Assembly) and CROC. (PDF) [file pgen.1012162.s010.pdf]

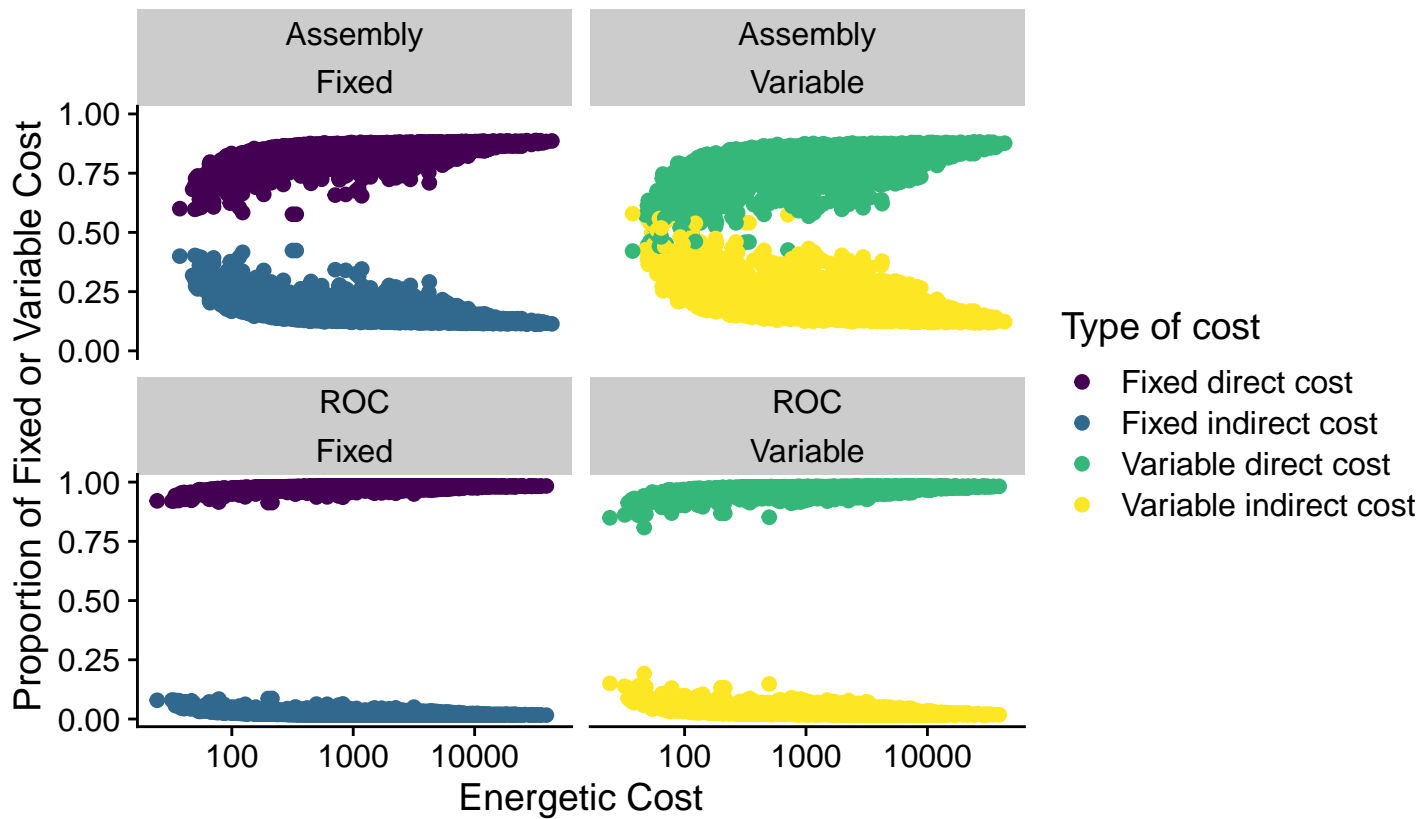

Supplement: S10 Fig — Comparison of proportion of total fixed or variable cost per gene as a function of total energetic flux (cost times the protein production rate) based on Ct1/2 (Assembly) and CROC. (PDF) [file pgen.1012162.s011.pdf]

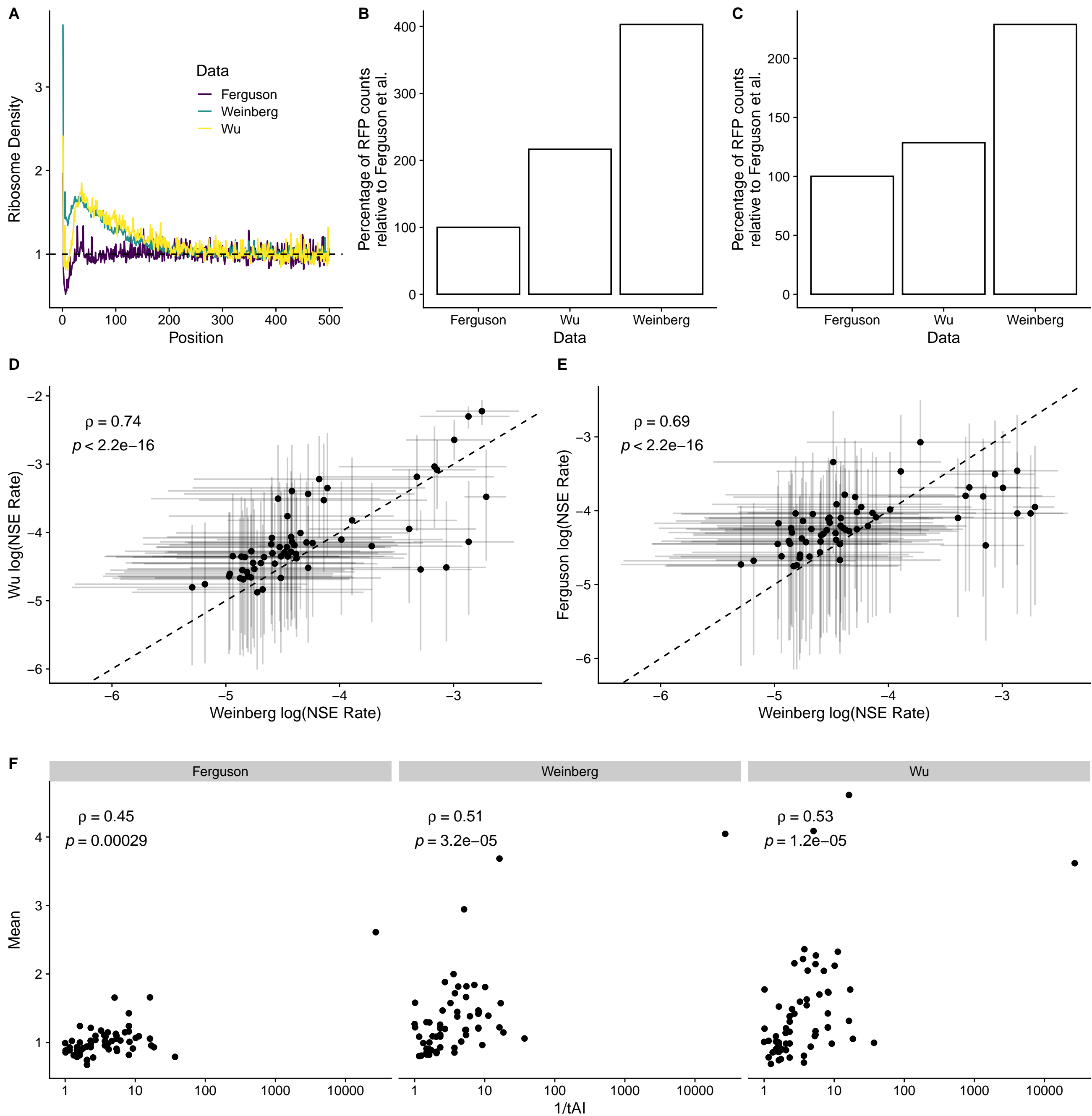

Supplement: S11 Fig — (A) Comparison of metagene ribosome densities from Weinberg et al. [27], Wu et al. [29], and Ferguson et al. [53]. (B) Total number of ribosome footprints included in the final PANSE analysis for each dataset (excluding the 5’-ends), expressed as a percentage relative to the Ferguson et al. data. (C) Same as in (B), but only considering the CDSs included in the Ferguson et al. analysis. (D) Comparison of the NSE rate b estimates (on log10 scale) from the Weinberg et al. and Wu et al. datasets. Error bars represent the 95% HDIs. (E) Same as in (D), but using the Ferguson et al. dataset. (F) Comparison of PANSE estimated ribosome waiting times wc and the inverse of codon weights estimated via the tRNA adaptation index (tAI). (PDF) [file pgen.1012162.s012.pdf]
