# Supplementary material for: The importance of nonsense errors: Estimating the rates and implications of ribosome drop-off during protein synthesis
Source: PLoS Genet. 2026 Jun 9;22(6):e1012162. doi: 10.1371/journal.pgen.1012162 (PMC13271495; doi:10.1371/journal.pgen.1012162)
Supplement: Attachment — Submitted filename: PlosGeneticsComments.pdf [file pgen.1012162.s016.pdf]

The authors present a comprehensive and technically sophisticated modeling framework (PANSE) for codon-specific inference of both elongation rates and nonsense error (NSE) rates. Leveraging the inferred parameters, they argue that natural selection acts against NSE and that the cost of nonsense errors is comparable to ribosome pausing. The modeling and inference effort is clearly substantial and laudable. That said, I have several concerns about the robustness of the results and the novelty of the contribution, as detailed below.

Major Comments

1. PANSE Model

The central difference between PANSE and previous models such as TASEP is the explicit introduction of codon-specific NSE rates (b_c in Table 1), whereas earlier approaches effectively assumed codon-specific elongation rates with a constant NSE rate. While this extension is conceptually important, the empirical gain appears modest based on the DIC presented. As noted in the Supplement, estimates of b_c are noisy (see also Fig. 2A), raising questions about whether the added model complexity justifies the marginal improvement in fit.

A related concern is identifiability. Because codon-specific elongation rates (governed by α_c *and* λ_c in Table 1) and codon-specific NSE rates are jointly inferred, some correlation or confounding between them seems unavoidable. This complicates interpretation, making it difficult to determine whether observed patterns reflect real biology or artifacts of model structure.

2. Evidence for Selection Against Codon-Specific NSE

Figure 2B: The regression results presented as evidence for codon-specific NSE sometimes contradict biological expectations (e.g., codons with two stop codon neighbors showing lower rather than higher NSE).

Figures 5B and 6C: The increase in elongation probability (Pr(no NSE)) with codon position is attributed to codon-specific b_c. However, this probability is not solely determined by b_c, but is instead a joint function of elongation rates (inverse of elongation waiting time) and b_c. Thus, the trend cannot be confidently attributed to codon-specific NSE. If the main result is simply that elongation probability increases with codon position, this has already been reported (e.g., Gilchrist & Wagner 2006 and Gilchrist 2009 cited by the authors).

In addition, the interpretation assumes that any nonsense error, regardless of position, abolishes protein function. Truncations near the 3′ end are often relatively benign, which weakens the inference of strong selective pressure against NSE.

Figure 6: The finding that highly expressed genes tend to avoid NSE raises the same interpretability issue. It remains unclear whether the observed pattern is driven by codon-specific b_c or by the combined effects of codon specific elongation rates and b_c. Again,

prior work has shown that highly expressed genes have higher elongation probabilities (e.g., Gilchrist & Wagner 2006 and Gilchrist 2009 cited by the authors).

Minor Comments

1. Terminology: The manuscript sometimes uses "NSE" to mean codon-specific b_c, and other times to mean the joint effect of codon-specific elongation rates (α_c *and* λ_c) and b_c. This dual usage is confusing and should be clarified consistently throughout.

Recommendations

Based on the above, I recommend acceptance pending revision. Specifically, the authors should:

1. Clarify whether inference of codon-specific elongation rates and codon-specific NSE rates is subject to spurious correlation, and discuss the implications for interpretation.

2. Reconsider the placement of results. If authors cannot show or clarify codon-specific NSE does not play a substantial role in Figures 5B and 6 after revision, these should be moved to the Supplement as confirmatory analyses. The main text should instead highlight newer contributions, such as those in Figures 2, 5A, and 7. For clarity and conciseness, Figures 3 and 4 (and their associated text) could also be relegated to the Supplement.

3. Be explicit about when "NSE" refers to b_c versus when it refers to the combined effect of elongation and nonsense error rates.
