# Supplementary material for: The importance of nonsense errors: Estimating the rates and implications of ribosome drop-off during protein synthesis
Source: PLoS Genet. 2026 Jun 9;22(6):e1012162. doi: 10.1371/journal.pgen.1012162 (PMC13271495; doi:10.1371/journal.pgen.1012162)
Supplement: Attachment — Submitted filename: response_to_reviewers.docx [file pgen.1012162.s018.docx]

We thank the reviewers for their insightful comments on our manuscript. Based on the reviewers' comments, we have added many additional or expanded analyses that we believe improve the overall confidence in our results. Our comments and responses are denoted by a dash ("-") symbol.

\# Reviewer 1:

1. Translating is the most error-prone step in the central dogma. Translation errors, such as amino acid misincorporation and stop codon read-through, as well as their evolutionary and functional implications, have been extensively studied. In this manuscript, Cope, Pak and Gilchrist studied yet another type of translation error, nonsense errors (NSEs), defined as pre-stop codon drop-offs of translating ribosomes. A computational model was developed that infers various key parameters of translation dynamics, including, most importantly, rates of NSE. For each codon, they estimated NSE rates by fitting their model to one ribosome profiling dataset from yeast. It is argued that NSE is a form of cellular burden similar to ribosome pausing, as a result of which it is selected against. Given the recent discovery of the biological significance of translation errors, I believe the authors have proposed an intriguing and meaningful biological hypothesis.

- We appreciate the reviewer's agreement that this is an important and relevant topic

2. The analyses presented are, however, far from convincing, especially with regard to the reliability of the NSE rate and the lack of necessary controls with respect to the argued natural selection. Below are my comments in more detail.

- We appreciate the reviewer's frankness, but disagree with several important points and their overall conclusion as we explain below.

3. The first and most important concern is the fact that the authors never really tested whether their NSE rate is (relatively) accurate. There may be no actual data that directly measure NSE, but the authors should at least try some data from indirect or manipulative experiments in order to demonstrate the reliability of their model on inferring NSE rates.

- We are not experimentalists and believe this is an unreasonable request. We do cite the limited experimental work that has been done on this topic in the Introduction and Discussion. As we note in the Discussion, our estimates are well in line with other NSE estimates in the literature.

- However, we have updated our regression (Figure 2) relating codon properties to NSE rates b_c to use 2 new properties that are suspected to lead to nonsense errors: empirical missense error probability (taken from Landerer et al. MBE 2024) and frameshift competency (taken from Vimaladithan and Farabaugh Molecular and Cellular Biology 1994). We also dropped the nucleotide identity at each codon position and the amino acid properties because these regressions had large Variance Inflation Factors (VIFs > 5), making the p-values unreliable. Mismatches in the codon-anticodons base pairings can destabilize the peptidyl-tRNA to allow for peptidyl-tRNA drop-off (Caplan and Menniger Journal of Mol. Biol. 1979) or frameshifts (Sundararajan et al. Mol. Cell 1999), the latter of which often leads to a nonsense error. For peptidyl-tRNA drop-off, recent work in E. coli suggests that the drop-off often occurs almost immediately after the missense error (Nagao et al. Nat. Comm. 2023). Along these lines, we observe that missense error rates and the frameshift competency of a codon increase NSE rates b_c (on the log10-scale) by 0.40 and 1.24 units, respectively (both have p < 1E-03). Given that the missense error probabilities are obtained from multiple high-throughput mass spectrometry experiments and can also be noisy, we suspect this coefficient is attenuated. The impact of having a stop-codon neighbor at the third position remains positive and statistically significant. Although these do not confirm our NSE rate b_c estimates, the fact that our estimates are positively correlated with other errors that often lead to nonsense errors suggests our estimates are picking up on these signals. We emphasize that our model is agnostic to the specific mechanisms causing nonsense errors.

4. I can think of a few possible directions.

i. The inferred NSE rate for a given codon should increase upon the KO/KD of some tRNA.

- Given our model formulation, we do not expect altering tRNA abundances to directly alter NSE rates, we do expect this to alter the NSE probabilities by increasing ribosome waiting times. As a result, we don't see how this analysis would further validate our estimates of NSE rates.

ii. For RNA editing events that result in premature stop codons, the NSE rate should presumably be similar to the fraction of edited mRNAs.

- To the best of our knowledge, RNA editing in mRNAs is rare or non-existent in S. cerevisiae (personal communication with Dr. Daohan Jiang (OIST)). The only paper we found relevant to general mRNA editing in S. cerevisiae was Wang et al. Genome Research 2016, which has been cited 22 times as of Jan. 2026. We note that previous work from this group using a similar approach in humans received significant public pushback from the scientific community (e.g., Pickrell et al. Science 2022). Given this and the lack of citations for a seemingly big discovery, we suspect researchers in the RNA editing field remain skeptical. Even so, if we take all of the RNA editing sites identified in the yeast strains considered by Wang et al., there would 1764 edited nucleotides potentially relevant to our analysis (i.e, excluding any N>C edits and genes not included in our analysis). These 1764 potential RNA edited sites make up ~0.1\% of codons in our dataset. We did not verify the frequency of these edits that result in a premature stop codon, but given the structure of the genetic code, it seems unlikely that it would be the majority. Importantly, the analysis by Wang et al. concluded most identified RNA edit sites are present in a small fraction of their respective mRNAs (median levels of 0.07). Taken together, we think fulfilling this request would necessitate analyzing new species, which is outside the scope of this current work.

iii. Treat the actual stop codons as sense codons, since they have different termination efficiency/readthrough probability, the model should be able to capture this difference.

- This is an interesting idea we have entertained ourselves as a means of estimating ribosome stop codon read through error rates. However, we do not see how this would add any meaningful validation of our model at the coding sites. Indeed, given the drop off in ribosome density after the vast majority of stop codons is zero, we can confidently predict that any nonsense error rate at these stop codons must be orders of magnitude greater than the 'elongation' (i.e. stop codon read through) rates.

(iii) Splicing or transcription termination errors might result in truncated mRNA. Perhaps corresponding RNA-seq can show a fraction of truncated mRNA, which should be matched by ribo-seq-estimated NSE.

- While we agree including additional biological realism such as truncated mRNAs could improve our model fit. We would like to point out that we do conduct a test of model adequacy and show our simple, yet incomplete model, adequately captures the behavior of the real data (see Figure 3a) We would like to remind the reviewer that all models are wrong in the sense that all models are incomplete. In addition, essentially all of the ribosome footpring studies we are aware of ignore the role of truncated mRNA on ribosome footprints. As a result, while we believe this is a biologically interesting point, we also feel it is well beyond the scope of our study.

5. I feel uneasy about the fact that the authors only focused on one ribo-seq dataset from 2016. It is possible to analyze much more ribo-seq data. Presumably, more recent datasets should be of higher quality, and at the very least higher sequencing throughput. As well, there has been other recently identified bias in traditional ribo-seq protocols, such as "Common cell lysis procedures distort ribosome profiling analyses of gene expression" (Could you please comment on this, and whether it impacts your conclusion?). The analysis of many more datasets, in many more species, is recommended.

- Although we have no reason to suspect there are issues with the Weinberg data (and there are reasons to believe it may be more reliable than even some newer datasets, see Mok et al. bioRxiv 2023), we applied our model to additional yeast datasets from Wu et al. Mol. Cell 2019 and Ferguson et al. Nat. Methods 2023. We find that NSE rate b_c estimates of these two datasets were correlated with the Weinberg et al. NSE rate estimates (Spearman rank correlations for 0.74 and 0.69 for Wu et al. and Ferguson et al. respectively), despite differences in the experimental protocols, the distribution of read lengths used (20-22nt and 28-30nt for Wu et al., 30-37nt for Ferguson et al, ribosome A-sites in this case were based on riboWaltz), and the genes included in the final analysis (same filtering criteria was applied to all 3 datasets). However, the codon-specific NSE rate estimates from the Ferguson et al. data were clearly noisier than either of the other two datasets, as indicated by the 95\% HDIs. We note that the Ferguson et al. data had far fewer read counts used in the PANSE analysis (approximately half the number of reads from Wu et al., approximately a quarter of the number of reads from Weinberg et al.). An analysis assuming NSE rates are the same for each codon provided a posterior mean value close to estimate from the same analysis performed using the Weinberg et al. dataset (Log10(NSE Rate) -4.03 for Ferguson vs. -3.85 for Weinberg.). We have added a subsection under Results titled "Parameter estimates across S. cerevisiae ribosome profiling datasets are largely consistent" that describe these results.

6. I believe that almost all patterns concerning NSE rate (its variation, selection, etc.) can be explained by codon optimality, as if NSE occurs less often in optimal codons than in non-optimal codons. The question arises as to how the authors can determine whether their observed patterns are actually due to selection against translational burden due to NSE, or whether they are due to other factors, such as tRNA misreading rates or translational efficiency (or ribosome pausing, as the authors mention). High-expression genes, for example, are known to use optimal codons more frequently.

- Because the sequences are given and because our model explictly accounts for variation in codon pausing times, our results do not rely on any assumptions about the role of selection, muation, or drift on codon usage bias (CUB). Unlike much of our other work, this model does not rely on evaluating how well adapted a given sequence is relative to a null model of mutation and drift. Instead, it simply infers elongation and NSE rates based on a gene's given sequence and observed ribosome densities. Contextualizing our estimate indicates that the strength of selection on CUB likely depends on both codon specific ribosome elongation rates and codon specific ribosome drop off rates. Given the reviewer's comment on this topic, we have added the following text to the discussion emphasizing these facts.

"We emphasize that our results are consistent with natural selection against nonsense errors, but our parameters do not reflect evolutionary parameters of interest in population genetics studies (e.g., selection coefficients). The most notable pattern of natural selection against nonsense errors is the enrichment of codons with higher NSE probabilities Pr(NSE) in the 5'-end due to presumably weaker selection shortly after translation initiation. As natural selection against nonsense errors is correlated with natural selection for efficient translation, these two selective pressures are correlated across genes, but the latter is not expected to lead to the enrichment of NSE-prone codons in the 5'-end \citep{Cope2018}."

7. In addition, Koonin and colleagues have demonstrated that the 5' end of a gene is more susceptible to other types of errors (e.g. frame shift) than the 3' end. According to them, errors at the 5' end are less costly than those at the 3' end, thereby suggesting that selection is taking place. The authors of this manuscript would benefit from doing a better job controlling these alternative hypotheses or at least explaining why the patterns they showed are not confounded by these other factors.

- We appreciate the authors concern that changes in NSE rates might be greatly elevated at the 5' end of most CDS. We are also concerned that ribosome elongation rates might be greatly reduced in this region as well. We even speculate in the paper that some combination of an increase in NSE rates b_c and a decrease in elongation rates are likely responsible for the rapid decline in ribosome density in the first 200 codon; a trend observed across ribosome footprinting datasets. Indeed, we chose to excluded the first 200 codons from our analysis to avoid the effect of these model violations on our model fitting. Regarding the observed codon usage patterns in the data, a challenge here is that frameshifts are (a) also related to speed of the ribosome elongation and (b) can contribute to ribosome drop-off. As our approach is agnostic to the causes of ribosome drop-off, these effects likely contribute to the NSE rates b_c estimated, much like the impact of missense errors. We have further elaborated on these points in the Discussion section with the following paragraph (also addresses Reviewer 1, Comment 6).

"Other selective pressures are hypothesized to lead to unique patterns of codon usage at the 5'-end of CDSs. A key challenge in understanding selection on synonymous codon usage is the potential for correlated selective effects. First, as previously discussed, is the ``5'-ramp hypothesis.'' In this case, the enrichment of slow codons, which generally have higher NSE probabilities Pr(NSE), could lead to a similar pattern observed for selection against nonsense errors. However, our previous work using a population genetics model to quantify natural selection shaping codon usage in \textit{S. cerevisiae} and \textit{E. coli} showed that the enrichment in slow codons in the 5'-end is more consistent with a reduction in the strength of natural selection for fast codons, rather than selection generally favoring slow codons in this region \cite{Cope2018,Cope2022quantifying}. Second, codon usage at the 5'-end is hypothesized to be shaped by selection against mRNA secondary structure to promote efficient translation initiation \citep{Goodman2013,Kudla2009}. However, evidence in \textit{S. cerevisiae} and other species suggests this effect is primarily relevant to the first 10-15 codons \citep{Gu2010}, whereas we see a gradual decrease in the probability of an NSE over a far broader region in the 5'-end. Third, previous work hypothesized natural selection against ribosomal frameshifting would be stronger at the 5'-end \cite{Huang2009}. Ribosomal frameshifts often result in premature translation termination due to encountering an out-of-frame stop codon soon after the frameshift \citep{Clarke1982,Seligmann2004,Itzkovitz2007} and can result from the presence of slow codons in the ribosome A-site in combination with a near- or non-cognate codon-anticodon match in the P-site \citep{Vimaladithan1994,Sundararajan1999,Huang2009}. We expect selection against ribosomal frameshifts to be strongly correlated with selection against nonsense errors. Along these lines, codons prone to causing frameshifts when located in the P-site \citep{Vimaladithan1994} had higher NSE rates \bc. Future work will focus on quantifying the evolution of codon usage bias as it relates to selection against nonsense errors. "

8. The model did not consider No-Go Decay (NGD) or non-sense mediated decay (NMD). I am not quite certain whether NSE will trigger NGD/NMD, and I am not certain why not accounting for NMD makes NSE rate estimation conservative (Line 574-575).

- Our reference to the conservative nature of our estimates was in terms of the bioenergetic cost (in terms of NTP) of an NSE. The NGD and NMD mechanisms require the activation of additional mechanisms that, if triggered by an NSE event, will require additional NTP not accounted for in our calculations. Again, we emphasize that all models are incomplete. During data filtering, we removed genes with codons showing large ribosome densities relative to the rest of the gene, which we suspect are potential targets for NGD.

7. Line 27-30. Does the "simple exponential waiting time model" refer to the authors' own work in the manuscript? Or the work of others? In either case, please make it clear.

- We apologize for this confusion and have revised our wording as follows to clarify we are assuming waiting times at a codon follow an exponential distribution.

"Each ribosome elongation step can be thought of as having two possible outcomes: a nonsense error or successful elongation. Assuming the amount of time a ribosome spends waiting before either events is exponentially distributed, the probability of a nonsense error at a given codon depends on both its nonsense error rate (i.e., the background rate at which a nonsense error event is triggered) and its elongation rate."

9. Do the releasing factors behave similarly to the tRNA (in terms of thermodynamics or kinetics of diffusion to the ribosome A site)? This needs to be clarified.

- We thank the reviewer for raising this question. Given the physical similarities between release factors and charged tRNAs, we expect they have similar thermodynamic and diffusion properties. However, given the fact that we do not use footprints at the stop codon, we make no explicit assumptions about the behavior of release factors in our model. While release factor properties such as diffusion likely affect codon specific NSE rates, the NSE rate estimates our model produces are agnostic with respect to their causes. We have added additional text throughout to clarify this point.

10. Line 91. According to this sentence, "b" represents elongation rate, whereas the next sentence suggests "c" represents elongation rate.

- We apologize for this typo. Indeed, "b" represents nonsense error rates and "c" represent elongation rates.

11. Line 111. It is necessary to provide support for the use of 15 nt offset, such as ribosome profiles near start and stop codons.

- We determined the 15nt offset to be adequate based on manual examination of such ribosme read distributions around the start and stop codon from the output of riboviz2, as well as comparisons of the waiting times estimated using these offsets to 1/tAI. Even so, we used the Bioconductor R package riboWaltz to estimate A-site offsets, with riboWaltz recommending 15nt offsets for read length 28, and 16nt offsets for read lengths 29 and 30. However, this had little impact on our parameter estimates (see S2 Fig.).

12. L130. Even finer-scale model fittings may be possible, such as assuming that the NSE rate is similar within some codon sites with similar elongate rates (decoding times). This may address differences in NSE between codons of the same type (assuming decoding time is a major determinant, which the author seems to agree with in L27-30).

- We whole heartedly agree, indeed the overlap in our 95% highest density intervals of our posterior distribution suggest as much. However, the goal of this work is to test whether NSE rates vary between codons, which our results strongly support. Finer-scale model fittings are beyond the focus of this work.

13. L270. Two citations are missing their parentheses.

- This typo has been fixed

14. L312. There is ambiguity in the phrase "the drop," as it could mean that the ribosome density is lower than elsewhere, but the authors are actually referring to a negative first derivative. Please rephrase it in a clearer manner.

- Thank you for pointing out our confusing use of terminology. We have changed it to "gradual decrease in ribosome densities along the first 200 codons" which we think better describes the observed pattern.

15. L364. Why is normalization performed in this manner? The cost of a nearly completed long protein is much greater than the cost of a nearly completed short protein. The length itself should be used without normalization in this logic.

- It seems to us that either a relative or absolute section could be justified. As a result, we have followed the reviewer's suggestion and performed our updated analysis as in Figure 5A (see below) using the first 100 codons, rather than the first 25% of codons. Our results show an overall similar conclusion, with an enrichment of codons with higher NSE probabilities in the 5'-end (Wilcoxon rank sum test p = 0.0011), but not for the 3'-end (Wilcoxon rank sum test p = 0.55).

16. L368. The test is not sufficiently specific. There is also a possibility that codons clustering around normalized position 0.5 may indicate that the codon appears only in the middle of the CDS and not at both ends. It appears that the authors are arguing for a near uniform distribution of those codons along the CDS.

- The reviewer is correct that this is a possibility. We updated this analysis to compare the frequency of each codon in the first 25\% of the gene with the ``middle'' (25\% - 75\%) of the gene, using the latter as the expected frequency we should observe in the first 25\% under the null of no significant difference in codon usage. We used a one-sided binomial test, specifically looking for codons enriched in the first 25\% of genes. Codons enriched in 5'-ends of genes generally had higher NSE probabilities (Wilcoxon rank sum test $p = 3.7E-08$), which would be the expectation under selection against nonsense errors. A similar test based on the last 25\% of the genes found no significant difference in NSE probabilities between codons enriched at the 3'-end and those that were not (Wilcoxon rank sum test p = 0.67). As noted above, we performed a similar analysis based on an absolute definition of the termini to be the first and last 100 codons (restricting this analysis to genes with a minimum length of 250 codons), finding consistent results.

\# Reviewer 2:

The authors present a comprehensive and technically sophisticated modeling framework (PANSE) for codon-specific inference of both elongation rates and nonsense error (NSE) rates. Leveraging the inferred parameters, they argue that natural selection acts against NSE and that the cost of nonsense errors is comparable to ribosome pausing. The modeling and inference effort is clearly substantial and laudable. That said, I have several concerns about the robustness of the results and the novelty of the contribution, as detailed below.

\## Major Comments

1. PANSE Model

The central difference between PANSE and previous models such as TASEP is the explicit introduction of codon-specific NSE rates (bc in Table 1), whereas earlier approaches effectively assumed codon-specific elongation rates with a constant NSE rate. While this extension is conceptually important, the empirical gain appears modest based on the DIC presented. As noted in the Supplement, estimates of b_c are noisy (see also Fig. 2A), raising questions about whether the added model complexity justifies the marginal improvement in fit.

- We understand the reviewer's concern about the noisy NSE rate estimates for most codons. Indeed, we suspect these estimates are more heavily influenced by our choice of prior. However, we do observe a few codons (e.g, TGG) that have higher b_c and narrower 95\% highest density intervals (HDIs). Based on these results, we hypothesize the ``best'' model would be one where most codons are assumed to have indistinguishable b_c, with a few codons being allowed to vary; however, this would take substantial work to implement. Information criterion like DIC are intended to help researchers decide if the additional complexity in the model is warranted. Our differences in DIC are well over 10, suggesting these additional model parameters are well supported and warranted.

A related concern is identifiability. Because codon-specific elongation rates (governed by \alpha_c and \lambda_c in Table 1) and codon-specific NSE rates are jointly inferred, some correlation or confounding between them seems unavoidable. This complicates interpretation, making it difficult to determine whether observed patterns reflect real biology or artifacts of model structure.

- The fact that our statistical analyses strongly support the model with these error rates supports their inclusion in the model. While we appreciate this concern, but we note that because we are using a Bayesian approach any issues with parameter correlation are reasonably addressed by looking at the posterior distributions which inherently are shaped by such correlations. Indeed just based on simple models of ribosome movement, NSE and elongations rates are likely mechanistically linked, suggesting the value of future model refinement, not our model's limitations.

2. Evidence for Selection Against Codon-Specific NSE

Figure 2B: The regression results presented as evidence for codon-specific NSE sometimes contradict biological expectations (e.g., codons with two stop codon neighbors showing lower rather than higher NSE).

- The ability to make inferences from data is inherently limited. In Figure 2B we list many plausable factors that could affect nonsense error rates. It seems unlikely that the coefficients for these are actually exactly 0.0000000. Instead, our data indicates that the noise in the data and the incopmleteness of our current model result in our inability to determine the sign of a given model coefficient (e.g `3rd position 2 stop neighbor`). A stop codon neighbor effect is only statistically significant for 1 stop codon neighbors. Alternatively there may be other biological factors in play. As we note in the Discussion section, "Taken together, our results indicate being a single nucleotide away from a stop codon at the codon's wobble position can increase the chances of an NSE, but it is not in and of itself sufficient to increase the NSE rates...Indeed, other mechanisms can trigger premature translation termination \citep{Zaher2011,Chiabudini2014,Presnyak2015,Chadani2017}.'' In our updated regression, we find that codons that are more prone to causing ribosomal frameshifts and those that have higher missense error probabilities have higher NSE rates b_c. Ribosomal frameshifts often result in out-of-frame stop codons, and mismatches between the codon and anticodon can lead to polypeptidyl-tRNA drop-off. Both of these are types of nonsense errors. After updating our regressions to remove significant multicollinearity (see Response to Reviewer 1, comment 3), we no longer observe a significant effect for stop codon neighbors at the 1st or 2nd position, which could be conceivably interpreted as being consistent with the fact that ribosomes are known to be more sensitive to 1st and 2nd position mismatches. This can be tested by a more detailed model of ribosome dynamics.

Figures 5B and 6C: The increase in elongation probability (Pr(no NSE)) with codon position is attributed to codon-specific b_c. However, this probability is not solely determined by bc, but is instead a joint function of elongation rates (inverse of elongation waiting time) and bc. Thus, the trend cannot be confidently attributed to codon-specific NSE. If the main result is simply that elongation probability increases with codon position, this has already been reported (e.g., Gilchrist & Wagner 2006 and Gilchrist 2009 cited by the authors).

- Thank you for bringing this issue to our attention. That was not our intention. Instead, our intention was to frame these results in the light of elongation and NSE rates. As a result, we have gone through these sections to ensure clarity regarding the difference between b_c and Pr(NSE), often through notation. Indeed, these conclusions have been observed before, but these were based on proxies for elongation rates and NSE rate estimates from targeted molecular biology studies. Although these results confirm previous results, these papers are 20 years old. Our hope is that this paper will spark renewed interest in understanding the impact of nonsense errors in shaping translation dynamics and coding-sequence evolution. As such, we believe it is valuable to show these results in the main text.

In addition, the interpretation assumes that any nonsense error, regardless of position, abolishes protein function. Truncations near the 3′ end are often relatively benign, which weakens the inference of strong selective pressure against NSE.

- We feel this is an absolutely reasonable concern. However, if sensitivity to nonsense errors drops off towards the end of a coding sequence, we would expect the signal for adaptation agains nonsense errors to be diminished, not amplified. In the future our model could be expanded to assume, and even estimate the parameters of, a sigmoidal relationship between protein completion and protein function.

Figure 6: The finding that highly expressed genes tend to avoid NSE raises the same interpretability issue. It remains unclear whether the observed pattern is driven by codon-specific b_c or by the combined effects of codon specific elongation rates and b_c. Again, prior work has shown that highly expressed genes have higher elongation probabilities (e.g., Gilchrist & Wagner 2006 and Gilchrist 2009 cited by the authors).

- See our response to the reviewer's comment on Figure 5B and 6C.

\## Minor comments

1. Terminology: The manuscript sometimes uses “NSE” to mean codon-specific b_c, and other

times to mean the joint effect of codon-specific elongation rates (\alpha_c and \lambda_c) and b_c. This dual usage is confusing and should be clarified consistently throughout.

- We have gone through the manuscript to clarify when we mean NSE as an event, codon-specific NSE rates b_c, and codon-specific NSE probabilites Pr(NSE) (i.e., the joint effects of elongation and NSE rates). Specifically, we have tried to keep to using the full term "nonsense error" when referring to the event, using b_c in conjunction with NSE rate, and Pr(NSE) in conjunction with NSE probabilitiy.

\## Recommendations

Based on the above, I recommend acceptance pending revision. Specifically, the authors

should:

1. Clarify whether inference of codon-specific elongation rates and codon-specific NSE rates is subject to spurious correlation, and discuss the implications for interpretation.

2. Reconsider the placement of results. If authors cannot show or clarify codon-specific NSE does not play a substantial role in Figures 5B and 6 after revision, these should be moved to the Supplement as confirmatory analyses. The main text should instead highlight newer contributions, such as those in Figures 2, 5A, and 7. For clarity and conciseness, Figures 3 and 4 (and their associated text) could also be relegated to the Supplement.

3. Be explicit about when “NSE” refers to bc versus when it refers to the combined effect of elongation and nonsense error rates.

- We thank the reviewer for recommending our paper for acceptance pending revision and giving specific recommendations to improve the overall quality of the manuscript. We hope you find our clarifications and responses satisfactory.

\# References

Caplan and Menniger, Journal of Mol. Biol. 1979, <https://doi.org/10.1016/0022-2836(79)90370-X>

Ferguson et al., Nat Methods 2023, <https://doi.org/10.1038/s41592-023-02028-1>

Landerer et al., MBE 2024, <https://doi.org/10.1093/molbev/msae048>

Mok et al., bioRxiv 2023, <https://doi.org/10.1101/2023.02.21.529452>

Nagao et al., Nat. Comm. 2023, <https://doi.org/10.1038/s41467-023-38077-5>

Pickrell et al., Science 2022, <https://doi.org/10.1126/science.1210484>

Sundararajan et al., Mol. Cell 1999, <https://doi.org/10.1016/s1097-2765(00)80229-4>

Vimaladithan and Farabaugh, Molecular and Cellular Biology 1994, <https://doi.org/10.1128/mcb.14.12.8107-8116.1994>

Wang et al., Genome Research 2016, <https://doi.org/10.1101/gr.207878.116>

Wu et al., Mol. Cell 2019, https://doi.org/10.1016/j.molcel.2018.12.009
