# Supplementary material for: The importance of nonsense errors: Estimating the rates and implications of ribosome drop-off during protein synthesis
Source: PLoS Genet. 2026 Jun 9;22(6):e1012162. doi: 10.1371/journal.pgen.1012162 (PMC13271495; doi:10.1371/journal.pgen.1012162)
Supplement: Attachment — Submitted filename: response_to_reviewers_auresp_2.docx [file pgen.1012162.s019.docx]

We appreciate the comments and suggestion from both reviewers. We believe they substantially improved the manuscript. We have incorporated some of their additional comments into the current version of the manuscript. Below, our responses are colored in blue.

\# Reviewer 1:

I would like to thank the authors for their response. I will detail my additional comments following the author's numbering of my original comments, since some of them have been miscommunicated.

3. I am not asking for additional experiments. All my previously proposed additional tests are feasible with existing related data instead of new experiments.

- We thank the reviewer for clarifying their point and continuing to push for these analyses. We believe these results will significantly strengthen confidence in the model.

4.i. “we do expect this to alter the NSE probabilities by increasing ribosome waiting times”. That is exactly what I meant. In my opinion, it is possible to examine the ribosome waiting times and NSE using public Ribo-seq data with tRNA abundance manipulated.

- We are unaware of any recent tRNA gene deletion ribosome profiling measurements in S. cerevisiae, but deletions of tRNA modifications enzymes are known to increase the waiting times of certain codons. We figured this was in a similar spirit to the reviewer's suggestion, as these modification knockouts might significantly impede the translation of specific codons. To test this, we used one replicate from Chou et al. 2017 that deleted the gene encoding elp1, which is part of the Elongator Complex. Strains with elp1 knockouts are known to experience slower translation of codons AAA, CAA, and GAA. For comparison, we also analyzed one replicate of the the wild-type strain. As expected, we saw decreased (increased) posterior mean elongation rates (waiting times) of the AAA, CAA, and GAA codons, but relatively little change in posterior mean NSE rates (they were all biased downward, but the undertainty for these NSE rates was high for these codons was large). This confirms that factors suspected to impact waiting times have a stronger effect on waiting times rather than the NSE rate estimates. These results are in the new S4 Fig(B,C). We have added the following paragraph to the Results section.

"As an additional test of PANSE's robustness, we tested if the deletion of the gene encoding a non-essential tRNA modification enzyme \textit{elp1} impacted primarily codon-specific elongation rates $\elongRate$ or NSE rates $\bc$. The deletion of \textit{elp1} is known to decrease the elongation rates of codons AAA, CAA, and GAA \citep{Nedialkova2015,Chou2017}; thus, we expect this effect to be primarily absorbed into the elongation rates $\elongRate$ parameter. By comparing elongation rates between the deletion strain and a wild-type strain \citep{Chou2017}, we observed a clear increase in elongation rates for the specified codons, but not an increase in NSE rates (\nameref{sfig:stop_codon_chou_etal}B,C)."

4.iii. I am not proposing a comparison between actual stop codons and NSE at coding codons. My proposal is to compare the three stop codons, whose "NSE" (read-throughrate) is known to differ. A good example of this is UGA, which has the highest read-through rate, so its "NSE" should be low.

- We modified the AnaCoDa R package to allow PANSE to estimate parameters for stop codons. Using the same genes as in the original Weinberg analysis, we included up to 15 codons following the stop codon if an empirical 3'-UTR was present (obtained from Mangkalaphiban et al. Plos Genetics 2021). We find that the stop codon TAA has the highest posterior mean NSE rate \b and stop codon TGA has the lowest posterior mean NSE rate \b. This aligns with the reviewer's expectation. These results are in the new S4 Fig(A). We have added the following paragraph.

" To the best of our knowledge, empirical measurements of codon-specific NSE rates $\bc$ are missing from the literature; however, the frequencies at which ribosomes mistakenly readthrough stop codons have been quantified using a variety of approaches \citep{Beznoskov2019,Mangkalaphiban2021}, with TAA and TGA being the least and most prone to readthrough, respectively. Stop codon readthrough results from a missense error at a ribosome awaiting a release factor, allowing it to elongate into the 3'-UTR of a transcript. We do not explicitly model the process of translation termination and rare missense error events at stop codons, but we expect stop codon ``NSE’’ rates $\bc$ to be anti-correlated with their readthrough efficiencies. By slightly modifying our Weinberg dataset to allow for up to 15 codons in the 3'-UTRs, we found that TAA had the highest ``NSE’’ rate $\bc$, while TGA had the lowest, consistent with independent estimates of stop codon readthrough efficiency (\nameref{sfig:stop_codon_chou_etal}A). All three stop codons had ``NSE’’ rates $\bc$ greater (by $\geq 2$ orders of magnitude) than the NSE rates of the sense codon.”

5. Good to see similar results from other datasets.

6 and 7. My point is that the alternative explanation of the pattern supporting the selection argument is not sufficiently excluded. But the added paragraph in Discussion seem to be good enough.

- We are glad the reviewer is satisfied with the added paragraph to the Discussion section.

12. It is not immediately clear to me that the goal of this work is to test whether NSE rates vary between codons. Indeed, only Fig1B/D , Fig2 and Fig6B are codon-centric, whereas other figures are more often codon site or gene-centric (although the authors could surely argue their gene-centric results are all based on their codon-distinguishing model), which I find more interesting. I suggest the authors clarify this major goal in the title. The authors may decide whether to take this suggestion or not.

- Thank you for your suggestion. It prodded us to revisit our title and revise it slightly.

15. Good to see that the conclusions are robust

16. Okay.

I am overall satisfied with other responses. I also noticed that there is a typo at L631. “Tthe”.

- We have fixed this typo. Again, we appreciate the reviewer’s comments and feedback on our manuscript.

\# Reviewer 2

The authors have addressed the issues I raised in a reasonable way. I have no further comments.

- We would like to again thank the reviewer for their helpful comments that we believe substantially improved the manuscript.
