# Supplementary material for: The importance of nonsense errors: Estimating the rates and implications of ribosome drop-off during protein synthesis
Source: PLoS Genet. 2026 Jun 9;22(6):e1012162. doi: 10.1371/journal.pgen.1012162 (PMC13271495; doi:10.1371/journal.pgen.1012162)
Supplement: Peer review history

# Peer review: Decision Letter 0

**DOI:** 10.1371/journal.pgen.1012162.r001

**Authors:** Kelly Dyer

5 Sep 2025

PGENETICS-D-25-00876

The Importance of Nonsense Errors: Estimating the Rate and Implications of Drop-Off Errors during Protein Synthesis

PLOS Genetics

Dear Dr. Cope,

Thank you for submitting your manuscript to PLOS Genetics. After careful consideration, we feel that it has merit but does not fully meet PLOS Genetics's publication criteria as it currently stands. Therefore, we invite you to submit a revised version of the manuscript that addresses the points raised during the review process.

Please submit your revised manuscript within 60 days Nov 04 2025 11:59PM. If you will need more time than this to complete your revisions, please reply to this message or contact the journal office at plosgenetics@plos.org. When you're ready to submit your revision, log on to https://www.editorialmanager.com/pgenetics/ and select the 'Submissions Needing Revision' folder to locate your manuscript file.

Please include the following items when submitting your revised manuscript:

* A rebuttal letter that responds to each point raised by the editor and reviewer(s). You should upload this letter as a separate file labeled 'Response to Reviewers'. This file does not need to include responses to any formatting updates and technical items listed in the 'Journal Requirements' section below.

* A marked-up copy of your manuscript that highlights changes made to the original version. You should upload this as a separate file labeled 'Revised Manuscript with Track Changes'.

* An unmarked version of your revised paper without tracked changes. You should upload this as a separate file labeled 'Manuscript'.

If you would like to make changes to your financial disclosure, competing interests statement, or data availability statement, please make these updates within the submission form at the time of resubmission. Guidelines for resubmitting your figure files are available below the reviewer comments at the end of this letter.

We look forward to receiving your revised manuscript.

Kind regards,

Jianzhi Zhang

Academic Editor

PLOS Genetics

Kelly Dyer

Section Editor

PLOS Genetics

Aimée Dudley

Editor-in-Chief

PLOS Genetics

Anne Goriely

Editor-in-Chief

PLOS Genetics

**Additional Editor Comments:**

Reviewer 1 raised substantial concerns, especially regarding reliability of the NSE rate and the lack of necessary controls. We would be reluctant to send your revised manuscript back to the reviewers unless these and other main issues from the two reviewers are adequately addressed.

**Journal Requirements:**

1) Please ensure that the CRediT author contributions listed for every co-author are completed accurately and in full.

At this stage, the following Authors/Authors require contributions: Alexander L Cope, Denizhan Pak, and Michael Gilchrist. Please ensure that the full contributions of each author are acknowledged in the "Add/Edit/Remove Authors" section of our submission form.

The list of CRediT author contributions may be found here: https://journals.plos.org/plosgenetics/s/authorship#loc-author-contributions

2) We ask that a manuscript source file is provided at Revision. Please upload your manuscript file as a .doc, .docx, .rtf or .tex. If you are providing a .tex file, please upload it under the item type u2018LaTeX Source Fileu2019 and leave your .pdf version as the item type u2018Manuscriptu2019.

3) Please provide an Author Summary. This should appear in your manuscript between the Abstract (if applicable) and the Introduction, and should be 150-200 words long. The aim should be to make your findings accessible to a wide audience that includes both scientists and non-scientists. Sample summaries can be found on our website under Submission Guidelines:

https://journals.plos.org/plosgenetics/s/submission-guidelines#loc-parts-of-a-submission

4) Please upload all main figures as separate Figure files in .tif or .eps format. For more information about how to convert and format your figure files please see our guidelines:

https://journals.plos.org/plosgenetics/s/figures

5) We have noticed that you have uploaded Supporting Information files, but you have not included a list of legends. Please add a full list of legends for your Supporting Information files after the references list.

6) Please ensure that the funders and grant numbers match between the Financial Disclosure field and the Funding Information tab in your submission form. Note that the funders must be provided in the same order in both places as well.

State what role the funders took in the study. If the funders had no role in your study, please state: "The funders had no role in study design, data collection and analysis, decision to publish, or preparation of the manuscript.".

If you did not receive any funding for this study, please simply state: u201cThe authors received no specific funding for this work.u201d

**Reviewers' comments:**

Reviewer's Responses to Questions

**Comments to the Authors:**

**Please note here if the review is uploaded as an attachment.**

Reviewer #1: Translating is the most error-prone step in the central dogma. Translation errors, such as amino acid misincorporation and stop codon read-through, as well as their evolutionary and functional implications, have been extensively studied. In this manuscript, Cope, Pak and Gilchrist studied yet another type of translation error, nonsense errors (NSEs), defined as pre-stop codon drop-offs of translating ribosomes. A computational model was developed that infers various key parameters of translation dynamics, including, most importantly, rates of NSE. For each codon, they estimated NSE rates by fitting their model to one ribosome profiling dataset from yeast. It is argued that NSE is a form of cellular burden similar to ribosome pausing, as a result of which it is selected against. Given the recent discovery of the biological significance of translation errors, I believe the authors have proposed an intriguing and meaningful biological hypothesis. The analyses presented are, however, far from convincing, especially with regard to the reliability of the NSE rate and the lack of necessary controls with respect to the argued natural selection. Below are my comments in more detail.

1. The first and most important concern is the fact that the authors never really tested whether their NSE rate is (relatively) accurate. There may be no actual data that directly measure NSE, but the authors should at least try some data from indirect or manipulative experiments in order to demonstrate the reliability of their model on inferring NSE rates. I can think of a few possible directions. (i) The inferred NSE rate for a given codon should increase upon the KO/KD of some tRNA. For RNA editing events that result in premature stop codons, the NSE rate should presumably be similar to the fraction of edited mRNAs. (iii) Treat the actual stop codons as sense codons, since they have different termination efficiency/readthrough probability, the model should be able to capture this difference. (vi) Splicing or transcription termination errors might result in truncated mRNA. Perhaps corresponding RNA-seq can show a fraction of truncated mRNA, which should be matched by ribo-seq-estimated NSE.

2. I feel uneasy about the fact that the authors only focused on one ribo-seq dataset from 2016. It is possible to analyze much more ribo-seq data. Presumably, more recent datasets should be of higher quality, and at the very least higher sequencing throughput. As well, there has been other recently identified bias in traditional ribo-seq protocols, such as "Common cell lysis procedures distort ribosome profiling analyses of gene expression" (Could you please comment on this, and whether it impacts your conclusion?). The analysis of many more datasets, in many more species, is recommended.

3. I believe that almost all patterns concerning NSE rate (its variation, selection, etc.) can be explained by codon optimality, as if NSE occurs less often in optimal codons than in non-optimal codons. The question arises as to how the authors can determine whether their observed patterns are actually due to selection against translational burden due to NSE, or whether they are due to other factors, such as tRNA misreading rates or translational efficiency (or ribosome pausing, as the authors mention). High-expression genes, for example, are known to use optimal codons more frequently. In addition, Koonin and colleagues have demonstrated that the 5' end of a gene is more susceptible to other types of errors (e.g. frame shift) than the 3' end. According to them, errors at the 5' end are less costly than those at the 3' end, thereby suggesting that selection is taking place. The authors of this manuscript would benefit from doing a better job controlling these alternative hypotheses or at least explaining why the patterns they showed are not confounded by these other factors.

4. The model did not consider No-Go Decay (NGD) or non-sense mediated decay (NMD). I am not quite certain whether NSE will trigger NGD/NMD, and I am not certain why not accounting for NMD makes NSE rate estimation conservative (Line 574-575).

5. Line 27-30. Does the "simple exponential waiting time model" refer to the authors' own work in the manuscript? Or the work of others? In either case, please make it clear.

6. Do the releasing factors behave similarly to the tRNA (in terms of thermodynamics or kinetics of diffusion to the ribosome A site)? This needs to be clarified.

7. Line 91. According to this sentence, "b" represents elongation rate, whereas the next sentence suggests "c" represents elongation rate.

8. Line 111. It is necessary to provide support for the use of 15 nt offset, such as ribosome profiles near start and stop codons.

9. L130. Even finer-scale model fittings may be possible, such as assuming that the NSE rate is similar within some codon sites with similar elongate rates (decoding times). This may address differences in NSE between codons of the same type (assuming decoding time is a major determinant, which the author seems to agree with in L27-30).

10. L270. Two citations are missing their parentheses.

11. L312. There is ambiguity in the phrase "the drop," as it could mean that the ribosome density is lower than elsewhere, but the authors are actually referring to a negative first derivative. Please rephrase it in a clearer manner.

12. L364. Why is normalization performed in this manner? The cost of a nearly completed long protein is much greater than the cost of a nearly completed short protein. The length itself should be used without normalization in this logic.

13. L368. The test is not sufficiently specific. There is also a possibility that codons clustering around normalized position 0.5 may indicate that the codon appears only in the middle of the CDS and not at both ends. It appears that the authors are arguing for a near uniform distribution of those codons along the CDS.

Reviewer #2: The review is uploaded as an attachment

**********

**Have all data underlying the figures and results presented in the manuscript been provided?**

Large-scale datasets should be made available via a public repository as described in the *PLOS Genetics*  data availability policy, and numerical data that underlies graphs or summary statistics should be provided in spreadsheet form as supporting information.

Reviewer #1: Yes

Reviewer #2: Yes

**********

PLOS authors have the option to publish the peer review history of their article (what does this mean?). If published, this will include your full peer review and any attached files.

If you choose “no”, your identity will remain anonymous but your review may still be made public.

**Do you want your identity to be public for this peer review?**  For information about this choice, including consent withdrawal, please see our Privacy Policy.

Reviewer #1: No

Reviewer #2: No

[NOTE: If reviewer comments were submitted as an attachment file, they will be attached to this email and accessible via the submission site. Please log into your account, locate the manuscript record, and check for the action link "View Attachments". If this link does not appear, there are no attachment files.]

**Figure resubmission:**

-->While revising your submission, we strongly recommend that you use PLOS’s NAAS tool (https://ngplosjournals.pagemajik.ai/artanalysis) to test your figure files. NAAS can convert your figure files to the TIFF file type and meet basic requirements (such as print size, resolution), or provide you with a report on issues that do not meet our requirements and that NAAS cannot fix.

After uploading your figures to PLOS’s NAAS tool - https://ngplosjournals.pagemajik.ai/artanalysis, NAAS will process the files provided and display the results in the "Uploaded Files" section of the page as the processing is complete. If the uploaded figures meet our requirements (or NAAS is able to fix the files to meet our requirements), the figure will be marked as "fixed" above. If NAAS is unable to fix the files, a red "failed" label will appear above. When NAAS has confirmed that the figure files meet our requirements, please download the file via the download option, and include these NAAS processed figure files when submitting your revised manuscript.

**Reproducibility:**

To enhance the reproducibility of your results, we recommend that authors of applicable studies deposit laboratory protocols in protocols.io, where a protocol can be assigned its own identifier (DOI) such that it can be cited independently in the future. Additionally, PLOS ONE offers an option to publish peer-reviewed clinical study protocols. Read more information on sharing protocols at https://plos.org/protocols?utm_medium=editorial-email&utm_source=authorletters&utm_campaign=protocols

Attachment Submitted filename: PlosGeneticsComments.pdf

# Author comment: Author response to Decision Letter 1

**DOI:** 10.1371/journal.pgen.1012162.r002

29 Jan 2026

Attachment Submitted filename: response_to_reviewers.docx

# Peer review: Decision Letter 1

**DOI:** 10.1371/journal.pgen.1012162.r003

**Authors:** Kelly Dyer

17 Feb 2026

PGENETICS-D-25-00876R1

The Importance of Nonsense Errors: Estimating the Rate and Implications of Drop-Off Errors during Protein Synthesis

PLOS Genetics

Dear Dr. Cope,

Thank you for submitting your revised manuscript to PLOS Genetics. Your manuscript was seen again by the original reviewers. While one of them is satisfied, the other is not. After careful consideration, we feel that it has merit but does not fully meet PLOS Genetics's publication criteria as it currently stands. Therefore, we invite you to submit a revised version of the manuscript that addresses the points raised during the review process.

Please submit your revised manuscript by Apr 18 2026 11:59PM. If you will need more time than this to complete your revisions, please reply to this message or contact the journal office at plosgenetics@plos.org. When you're ready to submit your revision, log on to https://www.editorialmanager.com/pgenetics/ and select the 'Submissions Needing Revision' folder to locate your manuscript file.

Please include the following items when submitting your revised manuscript:

* A letter that responds to each point raised by the editor and reviewer(s). You should upload this letter as a separate file labeled 'Response to Reviewers'. This file does not need to include responses to any formatting updates and technical items listed in the 'Journal Requirements' section below.

* A marked-up copy of your manuscript that highlights changes made to the original version. You should upload this as a separate file labeled 'Revised Manuscript with Track Changes'.

* An unmarked version of your revised paper without tracked changes. You should upload this as a separate file labeled 'Manuscript'.

If you would like to make changes to your financial disclosure, competing interests statement, or data availability statement, please make these updates within the submission form at the time of resubmission. Guidelines for resubmitting your figure files are available below the reviewer comments at the end of this letter.

We look forward to receiving your revised manuscript.

Kind regards,

Jianzhi Zhang

Academic Editor

PLOS Genetics

Kelly Dyer

Section Editor

PLOS Genetics

Aimée Dudley

Editor-in-Chief

PLOS Genetics

Anne Goriely

Editor-in-Chief

PLOS Genetics

**Reviewers' comments:**

Reviewer's Responses to Questions

**Comments to the Authors:**

**Please note here if the review is uploaded as an attachment.**

Reviewer #1: I would like to thank the authors for their response. I will detail my additional comments following the author's numbering of my original comments, since some of them have been miscommunicated.

3. I am not asking for additional experiments. All my previously proposed additional tests are feasible with existing related data instead of new experiments.

4.i. “we do expect this to alter the NSE probabilities by increasing ribosome waiting times”. That is exactly what I meant. In my opinion, it is possible to examine the ribosome waiting times and NSE using public Ribo-seq data with tRNA abundance manipulated.

4.iii. I am not proposing a comparison between actual stop codons and NSE at coding codons. My proposal is to compare the three stop codons, whose "NSE" (read-throughrate) is known to differ. A good example of this is UGA, which has the highest read-through rate, so its "NSE" should be low.

5. Good to see similar results from other datasets.

6 and 7. My point is that the alternative explanation of the pattern supporting the selection argument is not sufficiently excluded. But the added paragraph in Discussion seem to be good enough,

12. It is not immediately clear to me that the goal of this work is to test whether NSE rates vary between codons. Indeed, only Fig1B/D , Fig2 and Fig6B are codon-centric, whereas other figures are more often codon site or gene-centric (although the authors could surely argue their gene-centric results are all based on their codon-distinguishing model), which I find more interesting. I suggest the authors clarify this major goal in the title. The authors may decide whether to take this suggestion or not.

15. Good to see that the conclusions are robust

16. Okay.

I am overall satisfied with other responses. I also noticed that there is a typo at L631. “Tthe”.

Reviewer #2: The authors have addressed the issues I raised in a reasonable way. I have no further comments.

**********

**Have all data underlying the figures and results presented in the manuscript been provided?**

Large-scale datasets should be made available via a public repository as described in the *PLOS Genetics*  data availability policy, and numerical data that underlies graphs or summary statistics should be provided in spreadsheet form as supporting information.

Reviewer #1: Yes

Reviewer #2: None

**********

PLOS authors have the option to publish the peer review history of their article (what does this mean?). If published, this will include your full peer review and any attached files.

If you choose “no”, your identity will remain anonymous but your review may still be made public.

**Do you want your identity to be public for this peer review?**  For information about this choice, including consent withdrawal, please see our Privacy Policy.

Reviewer #1: No

Reviewer #2: No

[NOTE: If reviewer comments were submitted as an attachment file, they will be attached to this email and accessible via the submission site. Please log into your account, locate the manuscript record, and check for the action link "View Attachments". If this link does not appear, there are no attachment files.]

**Figure resubmission:**

-->While revising your submission, we strongly recommend that you use PLOS’s NAAS tool (https://ngplosjournals.pagemajik.ai/artanalysis) to test your figure files. NAAS can convert your figure files to the TIFF file type and meet basic requirements (such as print size, resolution), or provide you with a report on issues that do not meet our requirements and that NAAS cannot fix.

After uploading your figures to PLOS’s NAAS tool - https://ngplosjournals.pagemajik.ai/artanalysis, NAAS will process the files provided and display the results in the "Uploaded Files" section of the page as the processing is complete. If the uploaded figures meet our requirements (or NAAS is able to fix the files to meet our requirements), the figure will be marked as "fixed" above. If NAAS is unable to fix the files, a red "failed" label will appear above. When NAAS has confirmed that the figure files meet our requirements, please download the file via the download option, and include these NAAS processed figure files when submitting your revised manuscript.

**Reproducibility:**

To enhance the reproducibility of your results, we recommend that authors of applicable studies deposit laboratory protocols in protocols.io, where a protocol can be assigned its own identifier (DOI) such that it can be cited independently in the future. Additionally, PLOS ONE offers an option to publish peer-reviewed clinical study protocols. Read more information on sharing protocols at https://plos.org/protocols?utm_medium=editorial-email&utm_source=authorletters&utm_campaign=protocols

# Author comment: Author response to Decision Letter 2

**DOI:** 10.1371/journal.pgen.1012162.r004

27 Apr 2026

Attachment Submitted filename: response_to_reviewers_auresp_2.docx

# Peer review: Decision Letter 2

**DOI:** 10.1371/journal.pgen.1012162.r005

**Authors:** Kelly Dyer

11 May 2026

Dear Dr Cope,

We are pleased to inform you that your manuscript entitled "The Importance of Nonsense Errors: Estimating the Rates and Implications of Ribosome Drop-Off during Protein Synthesis" has been editorially accepted for publication in PLOS Genetics. Congratulations!

Before your submission can be formally accepted and sent to production you will need to complete our formatting changes, which you will receive in a follow up email. Please be aware that it may take several days for you to receive this email; during this time no action is required by you. Please note: the accept date on your published article will reflect the date of this provisional acceptance, but your manuscript will not be scheduled for publication until the required changes have been made.

Once your paper is formally accepted, an uncorrected proof of your manuscript will be published online ahead of the final version, unless you’ve already opted out via the online submission form. If, for any reason, you do not want an earlier version of your manuscript published online or are unsure if you have already indicated as such, please let the journal staff know immediately at plosgenetics@plos.org.

In the meantime, please log into Editorial Manager at https://www.editorialmanager.com/pgenetics/, click the "Update My Information" link at the top of the page, and update your user information to ensure an efficient production and billing process. Note that PLOS requires an ORCID iD for all corresponding authors. Therefore, please ensure that you have an ORCID iD and that it is validated in Editorial Manager. To do this, go to ‘Update my Information’ (in the upper left-hand corner of the main menu), and click on the Fetch/Validate link next to the ORCID field.  This will take you to the ORCID site and allow you to create a new iD or authenticate a pre-existing iD in Editorial Manager.

If you have a press-related query, or would like to know about making your underlying data available (as you will be aware, this is required for publication), please see the end of this email. If your institution or institutions have a press office, please notify them about your upcoming article at this point, to enable them to help maximise its impact. Inform journal staff as soon as possible if you are preparing a press release for your article and need a publication date.

Thank you again for supporting open-access publishing; we are looking forward to publishing your work in PLOS Genetics!

Yours sincerely,

Jianzhi Zhang

Academic Editor

PLOS Genetics

Kelly Dyer

Section Editor

PLOS Genetics

Aimée Dudley

Editor-in-Chief

PLOS Genetics

Anne Goriely

Editor-in-Chief

PLOS Genetics

www.plosgenetics.org

BlueSky: @plos.bsky.social

----------------------------------------------------

Comments from the reviewers (if applicable):

Reviewer's Responses to Questions

**Comments to the Authors:**

**Please note here if the review is uploaded as an attachment.**

Reviewer #1: My thanks go out to the authors for taking the time to fully address my comments in the two rounds of review. It is particularly pleasing and convincing to see the two additional analyses under point 4 to be completed and supportive for this round. Congratulations to the authors on their excellent work !

Signed, Jian-Rong Yang

**********

**Have all data underlying the figures and results presented in the manuscript been provided?**

Large-scale datasets should be made available via a public repository as described in the *PLOS Genetics*  data availability policy, and numerical data that underlies graphs or summary statistics should be provided in spreadsheet form as supporting information.

Reviewer #1: Yes

**********

PLOS authors have the option to publish the peer review history of their article (what does this mean?). If published, this will include your full peer review and any attached files.

If you choose “no”, your identity will remain anonymous but your review may still be made public.

**Do you want your identity to be public for this peer review?**  For information about this choice, including consent withdrawal, please see our Privacy Policy.

Reviewer #1: **Yes:** Jian-Rong Yang

----------------------------------------------------

**Data Deposition**

If you have submitted a Research Article or Front Matter that has associated data that are not suitable for deposition in a subject-specific public repository (such as GenBank or ArrayExpress), one way to make that data available is to deposit it in the Dryad Digital Repository. As you may recall, we ask all authors to agree to make data available; this is one way to achieve that. A full list of recommended repositories can be found on our website.

The following link will take you to the Dryad record for your article, so you won't have to re‐enter its bibliographic information, and can upload your files directly:

http://datadryad.org/submit?journalID=pgenetics&manu=PGENETICS-D-25-00876R2

More information about depositing data in Dryad is available at http://www.datadryad.org/depositing. If you experience any difficulties in submitting your data, please contact help@datadryad.org for support.

Additionally, please be aware that our data availability policy requires that all numerical data underlying display items are included with the submission, and you will need to provide this before we can formally accept your manuscript, if not already present.

----------------------------------------------------

**Press Queries**

If you or your institution will be preparing press materials for this manuscript, or if you need to know your paper's publication date for media purposes, please inform the journal staff as soon as possible so that your submission can be scheduled accordingly. Your manuscript will remain under a strict press embargo until the publication date and time. This means an early version of your manuscript will not be published ahead of your final version. PLOS Genetics may also choose to issue a press release for your article. If there's anything the journal should know or you'd like more information, please get in touch via plosgenetics@plos.org.

# Editor report: Acceptance letter

**DOI:** 10.1371/journal.pgen.1012162.r006

**Authors:** Kelly Dyer

PGENETICS-D-25-00876R2

The Importance of Nonsense Errors: Estimating the Rates and Implications of Ribosome Drop-Off during Protein Synthesis

Dear Dr Cope,

We are pleased to inform you that your manuscript entitled "The Importance of Nonsense Errors: Estimating the Rates and Implications of Ribosome Drop-Off during Protein Synthesis" has been formally accepted for publication in PLOS Genetics! Your manuscript is now with our production department and you will be notified of the publication date in due course.

The corresponding author will soon be receiving a typeset proof for review, to ensure errors have not been introduced during production. Please review the PDF proof of your manuscript carefully, as this is the last chance to correct any errors. Please note that major changes, or those which affect the scientific understanding of the work, will likely cause delays to the publication date of your manuscript.

Soon after your final files are uploaded, unless you have opted out or your manuscript is a front-matter piece, the early version of your manuscript will be published online. The date of the early version will be your article's publication date. The final article will be published to the same URL, and all versions of the paper will be accessible to readers.

For Research Articles, you will receive an invoice from PLOS for your publication fee after your manuscript has reached the completed accept phase. If you receive an email requesting payment before acceptance or for any other service, this may be a phishing scheme. Learn how to identify phishing emails and protect your accounts at https://explore.plos.org/phishing.

Thank you again for supporting PLOS Genetics and open-access publishing. We are looking forward to publishing your work!

With kind regards,

Judit Kozma

PLOS Genetics

On behalf of:

The PLOS Genetics Team

Carlyle House, Carlyle Road, Cambridge CB4 3DN | United Kingdom

plosgenetics@plos.org | +44 (0) 1223-442823

plosgenetics.org | Twitter: @PLOSGenetics
